# Water-Related Impacts of Climate Change on Agriculture and Subsequently on Public Health: A Review for Generalists with Particular Reference to Pakistan

**DOI:** 10.3390/ijerph13111051

**Published:** 2016-10-27

**Authors:** Toqeer Ahmed, Miklas Scholz, Furat Al-Faraj, Wajeeha Niaz

**Affiliations:** 1 Centre for Climate Research and Development, COMSAS Institute of Information Technology, Islamabad Campus, Park Road, Chak Shahzad, Islamabad 45550, Pakistan; toqeer.ahmed@comsats.edu.pk (T.A.); wajeehaniax@gmail.com (W.N.); 2 Division of Water Resources Engineering, Faculty of Engineering, Lund University, P.O. Box 118, Lund 22100, Sweden; f.a.m.al-faraj@edu.salford.ac.uk; 3 Civil Engineering Research Group, School of Computing, Science and Engineering, The University of Salford, The Crescent, Salford M5 4WT, UK

**Keywords:** bacteria, developing country, disease, environmental management, freshwater, pollution, sustainability, urbanization, wastewater, water resources

## Abstract

Water-related impacts due to change in climatic conditions ranging from water scarcity to intense floods and storms are increasing in developing countries like Pakistan. Water quality and waterborne diseases like hepatitis, cholera, typhoid, malaria and dengue fever are increasing due to chaotic urbanization, industrialization, poor hygienic conditions, and inappropriate water management. The morbidity rate is high due to lack of health care facilities, especially in developing countries. Organizations linked to the Government of Pakistan (e.g., Ministry of Environment, Ministry of Climate Change, Planning and Development, Ministry of Forest, Irrigation and Public Health, Pakistan Meteorological Department, National Disaster Management, Pakistan Agricultural Research Centre, Pakistan Council for Research in Water Resources, and Global Change Impact Study Centre), United Nation organizations, provincial government departments, non-governmental organizations (e.g., Global Facility and Disaster Reduction), research centers linked to universities, and international organizations (International Institute for Sustainable Development, Food and Agriculture, Global Climate Fund and World Bank) are trying to reduce the water-related impacts of climate change, but due to lack of public awareness and health care infrastructure, the death rate is steadily increasing. This paper critically reviews the scientific studies and reports both at national and at international level benefiting generalists concerned with environmental and public health challenges. The article underlines the urgent need for water conservation, risk management, and the development of mitigation measures to cope with the water-related impacts of climate change on agriculture and subsequently on public health. Novel solutions and bioremediation methods have been presented to control environmental pollution and to promote awareness among the scientific community. The focus is on diverse strategies to handle the forthcoming challenges associated with water resources management.

## 1. Introduction, Rationale, and Aim

Water-related disasters such as floods and droughts, changes in rainfall patterns, and increases in temperature are serious challenges, and their impacts on water quality and ultimately on human health have to be assessed and controlled [1]. About 50% of the recorded disasters are commonly due to floods and roughly 10% owing to high temperatures or heat waves [2]. Approximately 25% of all illnesses like diarrhea, malaria, and respiratory infection are due to change in climate and environmental degradation such as watercourse contamination from polluted agricultural runoff [3]. Challenges are particularly serious in developing countries. For example, according to the Global Facility for Disaster Reduction and Recovery, one-third of the people in Karachi lack clean water and health facilities [4].

With changing climate conditions such as rainfall, temperature, and humidity, water has great impacts both on agriculture and health. Water availability and quality are deteriorating and waterborne diseases are subsequently increasing, which impacts both sectors. Water quality in relation to climate change is not properly studied, especially the impact of climate change on water-related (both water- and vector-related) diseases. In Pakistan, waterborne diseases like cholera and vector borne diseases like malaria and dengue fever are prevailing with the changing rainfall pattern, temperature, humidity, etc. Water availability for agriculture is important as about 90% of water is used for irrigation purposes in many countries. With changing climatic conditions, water availability will affect agriculture and livestock production. There is a strong need to study these impacts with changing climatic scenarios.

**Figure 1:**
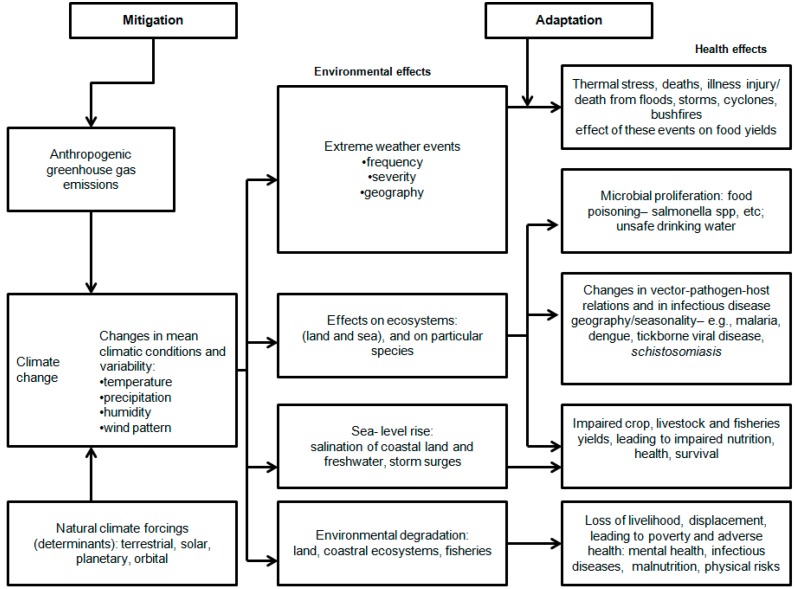
Overview of environmental and health effects linked to mitigation and adaptation strategies to climate change (after [5]).

Possible pathways through which climate change can affect multiple health outcomes are indicated in Figure 1. It follows that there is a need to assess water-related impacts of climate change on agriculture and subsequently on public health in general, but more particularly for developing countries, informing decision-makers with a wide range of professional backgrounds in environmental public health.

Various research institutes, academia, and government organizations are working on collaborative projects such as the projects “Impacts of Climate Change on Pakistan’s Water Sector Vulnerability Assessment” and “Glacial Lakes Outburst Floods” (funded by the United Nations Development Program). Other climate and water conservation projects are conducted by different government institutions such as the Climate Change, Alternative Energy, and Water Resources Department of Pakistan; Agriculture Research Center and Global Change Impact Study Center; as well as international non-governmental organizations. There are many other organizations both at government level and in the private sector working on impacts of climate change, natural disasters, and their direct impacts human health. Private and government authorities are also working on water quality and other water related issues.

There is currently neither a framework nor detailed analytical work that has been implemented showing the specific state of water resources management, agriculture, water quality, and public health with reference to climate change in Pakistan. Therefore, this paper aims to critically review scientific studies to assess the need for water conservation, risk management, and the development of mitigation measures to cope with the water-related impacts of climate change on agriculture and subsequently on public health, particularly from the Pakistani perspective. Novel solutions and strategies benefiting a wide range of generalists and stakeholders will be presented to control environmental pollution and to promote public awareness, and deliver the basis for a conceptual framework for the assessment of current and future scenarios.

## 2. Methodology

A comprehensive literature review was performed between January 2015 and June 2016. References were identified using Google Scholar (in addition to the standard Google search engine), Web of Science, Scopus, and Science Direct. The key words and phrases climate change, water-related impact, agricultural pollution, vector-borne disease, waterborne disease, flooding impact, water quality, extreme event, public health, pollution control, water availability, water quality, and Pakistan were used in various combinations. The publication time period considered was between 1980 and early 2016.

The literature cited was selected based on a critical review of publications of greatest relevance to Pakistan and countries with similar environmental boundary conditions. Other criteria such as publication date, outlet type, and author (or institution) played less important roles during literature selection.

## 3. Flooding Impacts and Environmental Pollution

Floods bring biological and chemical pollutants, which might ultimately contaminate the groundwater. Flooding events often cause soil erosion associated with chemical pollutants such as organic and inorganic matter, including heavy metals and polycyclic aromatic compounds, which pose threats to groundwater sources and may be taken up by crops and other plants [6]. Both fertilizers and flooded soils are sources of polycyclic aromatic hydrocarbons [6].

Water-borne and health-related infections may increase after flooding. An increase in mold after extensive flooding of healthcare facilities and a simultaneous decrease in multi-drug-resistant organisms has been noted [7]. Contamination with enteric Gram-negative bacteria (e.g., *Aeromonas* species), *Legionella* species, and non-tuberculous *Mycobacterium* species in hospital water sources has also been reported [7]. The spread of water-borne diseases like cholera and dysentery may increase after diarrhea outbreaks. The risk of dysentery might increase after flooding events [8]. Floods may increase both water-borne and vector-borne diseases such as cholera, typhoid, dysentery, and malaria as well as dengue and yellow fever [9]. Flood-related events increase the risk of infectious diseases, including respiratory and vector-borne illnesses [10].

The intensity of floods and droughts adversely affect public health during both the event and immediately thereafter. Climate change can increase the frequency and intensity of floods, droughts, and heat waves within a country or at a regional level, and their appearance is uncertain [11]. A summary of relatively recent flood losses in Pakistan is given in Table 1.

## 4. Water Quality and Public Health

Water pollution and associated public health implications belong to the most important challenges faced by humankind. Chemical (heavy metals such as lead, arsenic and iron), viral (hepatitis A, polio, and *Enteroviruses*) and bacterial (total and fecal coliforms along with *Streptococci*) pollution has profound effects on public health [12]. Various studies conducted in Bangladesh, China, and Pakistan reported on high concentrations of arsenic, but also on heavy metal pollutants like nickel, lead, chromium, cadmium, and mercury in groundwater, which directly affected public health [13,14]. One major source of water contamination is the improper disposal of sewage [15,16]. Both chemical and microbial pollution can affect aquatic life. Heavy metal pollution is a risk to fish, and subsequently to public health [17]. Improper management of sewage discharge and leakage of cesspool-influenced groundwater into coastal lagoons is responsible for some aquatic and environmental challenges [18,19].

Approximately 30% of the world population living in developing countries lacks access to sewage disposal [20]. *Escherichia coli* is often seen as the best indicator for fecal water pollution [21]. *Enteroviruses* are used as indicators to assess viral water contamination [22]. The diversity of antibiotic-resistant bacteria in aquatic environments can be enhanced by water pollution [23]. Heavy rainfall and floods associated with extreme weather and water supply contamination are responsible for waterborne disease outbreaks causing great risks for both developed and developing countries [24].

Climate change is often linked to an increase in occurrence of draughts and/or floods. Data relating floods with diarrheal patients in Dhaka (Bangladesh) during 1988, 1998 and 2004 have been discussed [25]. They reported that *Vibrio cholera* was the first and Rotavirus was the second most frequent cause of diarrhea. Other causes of diarrhea were enterotoxigenic *E. coli*, *Shigella*, and *Salmonella* species. Water-related pathogens cause diseases, their surveillance, and possible interventions are discussed in Table 2 [26,27,28]. No comprehensive published work has been reported on the relationship between water quality and fecal coliforms with special reference to climate change in Pakistan.

## 5. Overview of Agricultural Water Pollution Impacting on Human Health

This overview section is an introduction to this rather wide topic. Case study data usually depend on local and regional boundary conditions, which are often of little interest to the international reader. Therefore, the authors did not present numerical data, but provided a good number of references useful for readers requiring more information including data.

Agriculture can negatively affect water quality, especially in rural areas of both developing and developed countries [29]. Crops are often irrigated with wastewater polluted by domestic and industrial effluents containing heavy metals, which are potentially absorbed by vegetables and other cash crops, ultimately becoming part of the food chain. Literature [30] reported on potential toxicological risks of heavy metals on the public health using contaminated food. Urban agriculture has prominent effects on human health in terms of greenhouse gas emissions and spreading of water-related diseases such as malaria. Antonio-Nkondjio [31] pointed out that urban agriculture has a major role in spreading resistance to insecticides and malarial diseases. Chemical pollution can be minimized by applying natural fertilizers and sustainable agricultural principles in general.

Changes in temperature and rainfall due to climate change may influence the growth of fungi and increase in mycotoxins in agricultural food products, which are high risks to human health. Warm and humid climate is favorable for fungal growth where the average annual temperature is approximately 25 °C, but it varies depending upon season.

South Asian countries including Pakistan have been recognized as vulnerable regions subjected to the negative impacts of climate change in terms of public health [32]. A high population growth rate, deficiency in water availability, degradation of soil, urbanization, and an animal-based diet along with climate change are the global challenges, which are a threat to food security [33]. An increase in the demand for water in the agriculture sector for crops poses a severe threat to balanced water resources management. Only some crops such as wheat and barley are largely weather resistant, in particular, in a changing climate [34]. In general, the agriculture sector of most countries such as Pakistan has been adversely impacted by climate change, which may lead to a food shortage by 2030, resulting in an increase in food prices [35].

Droughts, floods, warming, and changes in precipitation can directly alter crop yields. This can threaten human life and cause long-term malnutrition and diseases. A study conducted on wheat production (Swat and Charsadda districts, Pakistan) suggests short duration and high yielding varieties should be introduced in hilly areas due to global warming [36].

Climate change has more profound impacts on agriculture and health as indicated in Table 3, which is based partly on literature and partly on expert opinion, because literature evidence is not conclusive in the interpretation of the strength of the case study relationships shown. Manure and animal waste contains pathogens, veterinary pharmaceuticals, and nutrients along with heavy metals, which are transported and spread into the environment, ultimately causing both environmental and human health hazards [37]. More intense storms due to climate change cause these contaminants to run-off and ultimately to enter the human food chain.

## 6. Water Pollution Control and Detection Techniques

This section follows the logic of the three previous sections: Section 3 links climate change to flooding, which impacts the environment, usually increasing water pollution. Section 4 indicates how water quality is linked to public health. Finally, Section 5 links more specifically agricultural water pollution to human health. Raw and agricultural water pollution and its impacts on human health are discussed in Section 6. Commonly used detection techniques and strategies developed by scientists to control the current problems and the need to develop new techniques under changing climatic conditions are assessed.

Bacteria-based systems can help in the degradation of environmental pollutants [38]. Phytoremediation removes toxic metals from wastewater. Empirical studies have reported on the use of plants for the removal of heavy metals and dyes from wastewater and soil [39,40]. Among the conventional technologies, various plant parts are used as adsorbents and natural coagulants as well as in advanced oxidation processes for the removal of metallic nanoparticles, toxic metals, and bacterial pollution from wastewater [41,42]. Whole cell sensing systems as well as chemical and biosensors are applied for the detection of environmental pollutants like metals. Recent advancements have shown the conversion of toxic metals into useful metallic nanoparticles by using microbes [43,44].

Biosensors have specific interactions between enzymes and substrate, and are highly specific in their detection capabilities. Bio-sorption is another emerging and novel technique for the removal of toxic metals from wastewater. Zouboulis et al. [45] used *Bacillus laterosporus* and *Bacillus licheniformis* for cadmium and chromium removal. They reported that bacteria have a high surface area-to-volume ratio like metallic nanoparticles, and that they therefore provide a large contact interface, which interacts with metals from the surrounding environment. Pyridine is one of the most widespread heterocyclic industrial contaminants. A new strain of *Arthrobacter* has been found to be the best among all the known strains for the purification of industrial wastewater [46]. *Arthrobacter*, which is used for the removal of atrazine, has good bioremediation potential concerning contaminated soils and waters [47].

Some solutions for the removal of environmental pollutants are conventional such as synthetic and natural coagulation, adsorption, and anaerobic digestion of contaminants. In comparison, others are advanced such as emerging nanotechnology-based techniques (e.g., the use of silver nanoparticles, titanium dioxide, zinc oxide, iron nanoparticles, and carbon nanotubes) for the removal of contaminants [48,49]. Both conventional and advanced solutions aim to provide and maintain better solutions to environmental pollution. Reducing contaminants can help in improving both environmental and human health. Improved housing design and better pipe material used in water supply can assist in reducing environmental pollution and improving public health in developing countries [50].

Animal waste-containing fecal matter can cause contamination to water resources. Anaerobic digestion of animal waste rather than dumping of waste at different places (as it is a common practice in developing countries) can cause environmental and public health problems [51]. Animal waste can also be used as an alternative source of energy. Therefore, anaerobic digestion of animal waste could be applied for the production of biogas, and different methanogens can enhance the process of digestion [37].

Emerging nanotechnology-based methods and nano-materials with their unique physical and chemical properties have previously been applied to reduce environmental pollution [49]. Silver and other metallic nanoparticles (e.g., zinc oxide) have been developed against waterborne pathogens [48]. Carbon nanotubes have been utilized as adsorbents, because of their unique properties beneficial for the removal of organic and inorganic pollutants along with radionuclides [52]. Results compared with natural zeolites indicated that the adsorbents are highly efficient and economical. Magnetic graphene oxides have been tested as adsorbates for the removal of trace metals of polybrominated diphenyl ethers in water treatment. They are regarded as highly efficient for environmental remediation [53]. Moreover, magnetic nano-composites are used as adsorbents for the removal of heavy metals such as chromium, lead, mercury, and arsenic [54].

## 7. Water Pollution, Climate Change, and Population Increase

Climate change is expected to alter water quantity and quality [55,56,57,58,59,60], and is likely to be different between large and small basins [61,62]. The potential changes in temperature and precipitation may not uniformly distribute over large watersheds [63] and thus both regional and local scales as well as the extent of urbanization must be considered in climate change impact studies for water resources protection [59]. Climate change has dynamic impacts on surface watercourses such as rivers, streams, and lakes, which provide clean water. In order to protect water resources, policy makers should consider climate change impacts on the reduction of pollution for improving water quality [59].

Human and industrial (including agriculture) wastes are the main pollutants contaminating water bodies particularly during flood events. Bacterial pollutants of human origin such as *E. coli*, *Salmonella*, and *Vibrio* as well as heavy metals and pesticide-related carcinogens are originating from different industries and are discharged into water bodies, ultimately causing pollution [64], which puts farming communities particularly at risk. Both bacteriological and chemical water pollutants have negative effects on human health, but less information is available in relation to changing climatic conditions. An increase in population and urbanization is putting water resources in danger, negatively affecting public health, the economy, and the environment [65].

Extreme climatic conditions such as high temperature and heavy precipitation play significant roles in increasing water pollution. Urban runoff to rivers and streams pollute water resources [66]. Different waterborne pathogens such as *Giardia* cysts and Cryposporidium oocysts correlate positively with rainfall [67]. Vector- and waterborne diseases are enhanced by El Niño-related extreme climatic conditions [67]. Extreme weather events and climate change have momentous impacts on the hydrological cycle [68].

For example, in a drought-prone region in the Sindh Province (Pakistan), inhabitants either have no access to freshwater or use brackish water for drinking purposes [12]. Similarly, in the Baluchistan Province (Pakistan), the groundwater level is falling at a rate of 3.5 m annually, and it is predicted that groundwater will soon be exhausted [69].

Rainfall increases the fecal pollution in rivers and freshwater resources, and elevates the bacterial risk [70]. Warm temperatures and extreme rainfall are often contributing factors to waterborne disease outbreaks, for example, in Canada [71]. Heavy rainfall and floods are associated with extreme weather and may account for 55% and 53% of waterborne outbreaks, respectively [23]. Extreme weather events and changes in patterns increase the risk of gastrointestinal diseases. For example, in Japan (1999 to 2007), about 7.7% and 2.3% of infectious gastroenteritis case increases per week were recorded for a 1 °C increase in the average temperature and for every 1% decrease in relative humidity, respectively [72].

Changes in climatic conditions, precipitation, humidity, and wind pattern occur due to both natural and anthropogenic activities, which have environmental effects like changes in environmental factors, environmental degradation, and disturbance of ecosystems. Changes in environmental conditions, disturbances, and degradations may affect human health negatively [5].

## 8. Pollution and Public Health

About two-fifths of the global population (including the people of Pakistan) are facing water scarcity and waterborne diseases. One child dies approximately every eight seconds, because of waterborne diseases [73]. Farooq et al. [74] estimates that 44% of the global population is unable to access clean drinking water. Ren et al. [75] conducted a study in the Huai River basin (China). They reported that preventive measures and interventions to support hygiene should be adopted to improve surface water quality.

A study conducted in Pakistan by Nabeel et al. [42] indicated that about 71% and 58% of the reported samples across the country were found contaminated with total and fecal coliform which causes loss of 25–58 billion Pak Rupee annually. Similarly, Ahmed et al. [76] reported on the presence of coliforms and fecal coliforms in 67% of water samples tested from academic institutions at different parts of Abbottabad city. This study highlighted the prevalence of water-related diseases like diarrhea and typhoid.

Chemical pollution can cause several health problems. High nitrate levels in the water can be one of the co-factors for several cases of water-related Mathemoglobinemia, a condition in which methemoglobin (an oxygen-carrying metalloprotein hemoglobin type) cannot bind oxygen [77]. Both constructive and destructive impacts of climate change influence agriculture and public health, but destructive impacts have more devastating effects that need to be addressed (Table 4). The added word “potential” in the heading of Table 4 indicates that there is not necessarily consensus among all scientists. Therefore, expert opinion of the authors to make a judgement on the available evidence has been expressed.

## 9. The Pakistani Scenario with Reference to Similar Countries

Water pollution is one of the major current problems affecting human health in Pakistan, because water quality is poorly monitored and managed [78]. Nabeela et al. [76] reported that 20% to 40% of all diseases are related to drinking water contamination resulting in annual economic losses between U.S. $0.25 and $0.58 billion.

In Pakistan, parallel water supply and sewage pipes currently lead to cross contamination and corrosion. In China, the situation is somehow similar to Pakistan where water pollution control, cross contamination between aquifers, and failure of the pipe network construction are the main concerns [14]. The public in Pakistan is unaware of pollutants such as iron, nitrates, and sulphates. Similarly, in Iran, 71% of the public are unaware of nitrates and 78% of people are largely ignorant of its corresponding health hazards [79].

Humid and warm conditions promote the growth of microbes, exaggerating water-related and waterborne diseases. In Pakistan, summers are longer than winters, and the temperature ranges between 30 °C and 45 °C in Islamabad as well as other cities of Punjab. The temperature in Pakistan is predicted to rise by about 3 °C by 2040, and between 5 °C and 6 °C by the end of this century [55,68,80]. Heavy rain and change in temperature and moisture conditions, especially in the monsoon season, encourage the spread of malaria, dengue fever, diarrhea, typhoid, and cholera [80]. Sterk et al. [81] studied the effect of climate on *Campylobacter* and *Cryptosporidium* under different climatic conditions and reported that climate change can increase the release but overall effect is limited.

Concerning Pakistan, the spread of dengue fever through viruses, which were imported into the country through tire trading and movement of travelers, is seen as a serious public health risk [82]. Climate change indirectly supports the growth of *Aedes aegyptei* larvae and adults, which cannot survive below 10 °C and below 5 °C, respectively [83].

Changes in temperature and humidity have disturbed the ecological equilibrium and contribute to new epidemics of malaria, dengue fever and other vector-related diseases [84]. There are a considerable number of studies correlating an increase in malaria with global warming. However, there is no sound evidence for this correlation [85]. Low temperature stops the growth of mosquitoes. An increase in temperature during the summer favors the growth of mosquitoes spreading malaria and dengue fever [84].

Dengue fever is endemic in Pakistan and spreads with an increase in temperature. As the temperature rises, the number of cases reported increase. Over-population as well as inadequate facilities in homes and towns, along with poor management of sewerage and water management, are the major reasons for the spread of dengue fever. Stagnant water in cities and towns provide breeding habitat for vector growth and its multiplication. Other reasons include travel and spread of viral infection from one region to another. Details of suspected cases, laboratory-confirmed cases, and deaths (confirmed by the World Health Organization in various unpublished reports) are given in Table 5.

Wang et al. [86] studied the variation in water quality in urban, suburban, and rural areas of Shanghai (China). They reported that well-managed urban areas have a better water quality compared to rural areas. Similarly, in Pakistan, rural areas have frequently poor water quality in comparison to the capital city Islamabad, as 90% of rural inhabitants lack access to clean water [87]. The country is facing plenty of challenges like a population boom, lack of natural resources, and no development of new reservoirs, which may lead to long-term water access per capita of less than 1000 m^3^. This water shortage may get more severe in regions which are located outside the Indus basin, where the average per capita water demand is already less than this threshold [88].

According to numerous international standards, there should not be any fecal coliforms in 100-mL of drinking water. In Pakistan, the main challenge is bacteriological contamination. The years 2007 and 2011 have been the worst years in regard to dengue virus infection in Pakistan [89]. The dengue virus circulates in Pakistan throughout the year with a peak incidence in the post monsoon period. Recent floods in Pakistan made the situation worse [90]. Mashiatullah et al. [91] reported that the feeding streams of Rawal Lake in Islamabad are contaminated by bacteria. The lake provides drinking water to more than 1.5 million inhabitants in Rawalpindi. Similarly, Ahmed et al. [92,93] reported on different dams located in the Pothwar region showing contamination of water resources with fecal matter.

## 10. Approaches to Diminish Water-Related Impacts

This section focuses on current health challenges. Specific approaches proposed by the authors to mitigate water-related impacts partly due to climate change for countries comparable to Pakistan include the following:

1. Reducing water scarcity and pollution by changing lifestyles of individuals through education and by applying water conservation strategies, rain water harvesting, integrated sustainable water management, as the global water requirement is expected to increase by 55% in 2050. (In Pakistan, the annual water availability was 5260 m^3^/capita in 1951. Due to increasing urbanization and anthropogenic activities, it is expected to be <800 m^3^/capita in 2030. In comparison, the estimated figure is 1066 m^3^/capita in 2015.).
2. Improving laws linked to the introduction of missing water valuation, surface water quality impacts due to climate change, groundwater challenges, and pollution control through awareness raising, and implementation of policies regarding water storage, water conservation, integrated water resources management, capacity building, and awareness raising that should be encouraged at various levels by introducing fines to reduce water scarcity and pollution problems should be encouraged [94,95].
3. Developing new and improved irrigation techniques and strategies (e.g., drip irrigation, site specific irrigation, micro-irrigation and sprinkling), crop rotation, introduction of new crop varieties, alternate crop selection [96], as well as applying innovative ideas for risk management, biodiversity enhancement, land and grazing management, water conservation, adaptation, and rehabilitation along with pollution control. Adaptation of strategies like crop management practices along soil fertility and water management are practiced due to a reduced availability of water and associated socio-economic effects [97]. Other strategies related to water management in agriculture include tunnel farming, alternate or renewable energy sources, especially pasteurized irrigation systems, gated piped irrigation, and laser land leveling [98].
4. Provision of more funds for research in biotechnology supporting the introduction of specific heat resistant, flood (or drought) tolerant, water efficient, and high yield crop varieties; adaptation of better strategies like water channels to conserve water by minimizing water evaporation and transpiration, water reuse strategies after consuming or using water, dam construction to enable water storage; minimizing the effects of water-related impacts of climate change on agriculture and health; and ensuring both water and food security [94]. Provision of funds to establish climate change research centers in agricultural departments to support highly efficient technologies to save labor and to increase yields along with optimized pollution control in the agriculture sector under changing climate scenarios is encouraged [95].
5. Involvement of national bodies and key local stakeholders, and securing their active engagement and contributions in adaptation and rehabilitation during and after disasters such as severe floods, earthquakes, heat waves, and storms. Educating farmers may be a considerable challenge when developing strategies to improve crop varieties and better breeds, which are tolerant to adverse climatic conditions like change in temperature, floods, and shift in rainfall patterns—as social barriers may limit the use of feasible measures by farmers [96,99].
6. Increasing transparent access to high quality information about water-related disasters and resulting impacts of climate change by national meteorology departments and disaster management control authorities along with provision of data for research-related projects to improve existing scenarios.
7. Increasing access to clean water sources subject to water quality assessments at specific periods, especially in flood-prone areas. This should be the top priority to avoid severe health challenges in flood affected areas.
8. Supporting the health system by improving surveillance and monitoring as well as proper disposal of sewage to avoid agricultural pollution, soil erosion, and sedimentation. Minimizing the impact and use of agriculture chemicals—like fertilizers, insecticides, pesticides, and leaching of other chemicals like toxic metals—on the enrichment of nutrients in ground water and its subsequent impacts on water quality and food security [100].
9. Improving health care facilities and availability of medicines and early responses to disasters to reduce casualties.

## 11. Recommendations for Further Work

To reduce the effects of climate change on health and agriculture, the following recommendations should be implemented:

1. To use this review as the basis for the development of a conceptual framework by which to consider the status of water-related impacts of climate change on agriculture and subsequently on public health in countries such as Pakistan.
2. To develop efficient and effective community-related projects to minimize the water-related impacts of climate change on agriculture and public health;
3. To build capacity and strengthen surveillance against waterborne disease outbreaks;
4. To enhance capacity and develop mechanisms for the diagnoses and control of water-related diseases;
5. To develop adaptation and mitigation strategies to improve the response to climate change-induced effects on health and agriculture;
6. To increase awareness among the general public through education, radio, TV programs, and print media;
7. To involve the community in the reduction of water and agriculture pollution, and to subsequently minimize the water-related infections originating due to climate change;
8. To develop an efficient drinking water quality monitoring scheme in order to mitigate water-related impacts of climate change and ensure a constant delivery of safe water;
9. To create assessment plans for the evaluation of community-related projects benefiting the public; and
10. To mobilize health professionals to protect the health and wellbeing of future generations via seminars, workshops, and training sessions for locals.

## 12. Conclusions

Climate change is likely to have a profound effect on public health, agriculture, and economy; especially on water-related impacts in terms of water quality, diseases, and changes in patterns of rain and droughts in some parts of the world including Pakistan. There is a strong link between agricultural pollution and public health.

This review highlights the need for water conservation, risk management, and the development of adaptation and mitigation strategies to cope with the water-related impacts of climate change. Different aspects regarding water-related impacts of climate change on health and agriculture have been discussed, and novel solutions and bioremediation methods have been presented to control environmental pollution and to promote public health awareness among the scientific community.

There is also a requisite to encourage regional and national authorities such as the Ministry of Climate Change and authorities working under the Ministry of Science and Technology and the Ministry of Food Security to intervene and to make changes in policies and laws with respect to climate change that affect the water, health, and agriculture sectors. Contradictory policy measures like electricity subsidies to farmers for tube wells in different provinces of Pakistan (especially in Baluchistan, where it is difficult to get water from aquifers due to low water tables) and loans for providing additional sugar mills, rather than improving existing ones, should be reconsidered. Introduction of heat resistant and flood (or drought) tolerant varieties as well as more yield- and water-efficient crop varieties, drought resistant grasses, and plants in urban areas (especially along the roadsides) should be introduced to conserve water. Development of better strategies like water channels to conserve water and dam constructions to enhance water storage are important to save Pakistan during both drought and flood events. The adaptation of more crops to the ‘less drops strategy’ to minimize the effects of water-related impacts due climate change on agriculture, water quality, and their subsequent health concerns should be encouraged.

## Figures and Tables

**Table 1:** Summary of estimated flood losses over the past few years in Pakistan.

Year	Deaths	House Damages	Population Affected
2010	490	1,602,800	175,000,000
2011	69,890	15,977,500	9,345,000
2012	570	636,400	4,849,800
2013	330	46,200	1,489,100
2014	370	107,100	2,412,000
2015	150	4800	1,314,500

**Table 2:** Overview of water-related pathogens (both waterborne and vector-borne), diseases, and their impacts.

Pathogen Types and Typical Organisms	Typical Major Common Diseases	Surveillance	Interventions	References
Waterborne pathogens (examples)				
*Salmonella* spp.; *Escherichia coli*; *Vibrio cholera*; *Shigella* spp.; *Streptococci*; *Yersinia* spp.	Typhoid; abdominal cramps; Dysentery; Cholera; sore throat; Yersiniosis	Microbiological water quality; monitoring, especially during and after a monsoon	Track diseases; monitor water quality and health; diagnose and investigate waterborne and other infections outbreaks; apply health interventions	[26,27]
Water-based pathogens (examples)				
Poliovirus; *Enteroviruses*; Rotavirus; Hepatitis A and E viruses; *Helicobacter pylori*	Polio; diarrhoea; stomach ulcers; infections; respiratory illnesses	Microbiological water quality monitoring during and after a monsoon	Track diseases; monitor health; diagnose and investigate waterborne outbreaks; apply health interventions	[26,27]
Vector-borne pathogens (examples)				
Sand flies; dengue virus (mosquitoes); Plasmodium (mosquitoes); Flavivirus; Tsetse flies	*Leishmaniasis*; dengue fever; fever with shivering; Japanese encephalitis; West Nile virus; Yellow fever; Trypanosomiasis	Monitoring of vectors and their habitats; source identification and enhanced investigations	Diagnose and investigate vector-borne and other related outbreaks; apply the health interventions available	[26,28]

**Table 3:** Potentially strong relationships between exposure situations and health conditions relevant to agricultural food production and storage (based on this literature review and the expert opinions of the authors).

Health Conditions of Concern	Polluted Air	Excreta and Household Wastewater	Polluted Water or Deficiencies in Water Management	Polluted Food	Unsuitable Housing	Global Change of Environment
Acute respiratory infection	X		X		X	
Other infections		X		X	X	
Diarrheal diseases		X	X	X		X
Malaria and other vector-borne diseases			X			X
Injuries and poisonings	X	X	X	X	X	X
Mental health conditions					X	
Cardiovascular diseases	X			X	X	X
Cancer	X					X
Chronic respiratory diseases	X					X

**Table 4:** Potential impact of major climatic elements on agriculture and health (based on this literature review and the expert opinions of the authors).

Climatic Elements	Potential Impacts			
	Constructive		Destructive	
	Health	Agriculture	Health	Agriculture
Drought	Decrease in vectors and vector-borne diseases	Parasites and vector growths	Water quality and availability is affected; water-related diseases and death increase; hunger	Loss of water; reduction in crop productivity; economic growth
Flood	-	-	Poor water quality; injuries; increase in vectors, water-related vector-borne and zoonotic diseases; heat stress; cardiovascular failure; anxiety; depression	Reduction in crop productivity and economic growth; infrastructure destruction
Heat and humidity	-	May increase crop productivity	Decrease in water availability; skin infection; increase in vectors and vector-borne diseases like malaria, dengue fever, and Japanese encephalitis	Increase in vectors and pests, and related agricultural problems and crop diseases
Salt water intrusion	Decrease in the growth of pathogens and related diseases	-	Water quality and supply degradation; diseases,	Agricultural land loss; increase in soil salinity; water supply and quality deterioration

**Table 5:** Dengue fever cases reported in Pakistan.

Year	Suspected Cases	Cases Laboratory-Confirmed	Deaths
2006	4961	1931	41
2007	2304	1226	18
2008	2792	2469	17
2009	1940	1085	13
2010	15,901	11,024	40
2011	252,935	17,057	219
2012	3913	639 (Karachi only)	-
2013	9037	8546	33
2014	-	504	6
2015	-	327 (Sindh)	-
